# Involvement of nitric oxide (NO) in cough reflex sensitivity between non-sensitized and OVA-sensitized guinea pigs

**DOI:** 10.1186/1745-9974-7-5

**Published:** 2011-09-22

**Authors:** Akihiro Hori, Masaki Fujimura, Noriyuki Ohkura, Akira Tokuda

**Affiliations:** 1 Respiratory Medicine, Cellular Transplantation Biology, Kanazawa University Graduate School of Medical Science, 13-1 Takara-machi, Kanazawa 920-8641, Japan

## Abstract

**Background:**

Exhaled nitric oxide (ENO) is elevated in bronchial asthma patients, and inhaled corticosteroid therapy lowers the elevated ENO levels in such patients. ENO appears to be an inflammatory marker, but its role in the pathophysiology of cough remains unclear. This study aimed to elucidate the relationship between NO and increased cough reflex sensitivity induced by allergic airway reactions.

**Methods:**

Cough reflex sensitivity to inhaled capsaicin was observed under NO depletion caused by NO synthase (NOS) inhibitors in non-sensitized and ovalbumin (OVA)-sensitized guinea pigs. The bronchoalveolar lavage fluid (BALF) was analyzed in an NO depletion setting using the inducible NOS (iNOS) inhibitor ONO1714 in OVA-sensitized guinea pigs.

**Results:**

NO depletion by the non-selective NOS inhibitor L-NAME suppressed cough reflex sensitivity in non-sensitized guinea pigs and OVA-induced increase in cough reflex sensitivity in sensitized guinea pigs; however, iNOS inhibition caused by ONO1714 partially suppressed the OVA-induced increase in cough reflex sensitivity, but not the normal cough response in non-sensitized guinea pigs. ONO1714 did not change BAL cell components in OVA-sensitized guinea pigs.

**Conclusions:**

The results suggest that NO may be involved not only in the normal cough reflex circuit, but also in the OVA-induced increase in cough reflex sensitivity, possibly via a different mechanism of action. Further studies are needed to clarify the precise mechanism.

## Background

Nitric oxide (NO) may play an essential role in regulating airway function and in the pathophysiology of inflammatory airway diseases [1]. NO is generated by NO synthase (NOS) from L-arginine in vivo [2]. NOS has three isoforms, namely neuronal NOS (nNOS: NOS-1), endothelial NOS (eNOS: NOS-3), and inducible NOS (iNOS: NOS-2) [2-6]. The former two isoforms are constitutive isozymes [7], and are assumed to regulate physiological homeostasis. The latter NOS can produce a much greater amount of NO than the constitutive forms [8]. High concentrations of NO may have not only beneficial functions (e.g. antibacterial, antiparasitic and antiviral), but also detrimental results, such as endotoxin shock [9], apoptosis [10], and pro-inflammatory effects [11,12].

Exhaled nitric oxide (ENO) is at significantly elevated levels in bronchial asthma patients compared to healthy subjects [13]. Immunostaining of biopsied bronchial mucosa has shown that iNOS is generally present in much more amounts in the bronchial epithelium of bronchial asthma patients than normal subjects [3]. Glucocorticoids inhibit expression of iNOS, but not of cNOS, in vascular endothelial cells [14]. These findings indicate that the augmentation of ENO results from increased iNOS expression in the airway of bronchial asthma patients. Recently, ENO measurements are recognized as a good surrogate marker for eosinophilic airway inflammation [15]. De Diego et al. reported that ENO levels in cough variant asthma patients were similar to those in bronchial asthma patients [16].

Increased cough reflex sensitivity to inhaled capsaicin has been reported in chronic cough associated with eosinophilic airway inflammation, such as non-asthmatic eosinophilic bronchitis [17], and atopic cough [18]. It is still controversial in bronchial asthma [19,20]. No study has investigated the relationship between cough reflex sensitivity and NO. In this study, we hypothesized that NO produced by iNOS might be a promoter in cough reflex sensitivity, and therefore performed the iNOS inhibition experiment using NOS inhibitors, in non-sensitized guinea pigs with normal cough reflex sensitivity and in OVA-sensitized guinea pigs which were of increased cough reflex sensitivity associated with allergic eosinophilic airway inflammation.

## Methods

### Animals

Male albino Hartley-strain guinea pigs (body weight, 450-500 g and 150-200 g), obtained from Sankyo Laboratory Service (Toyama, Japan), were used in the non-sensitized model experiment and in actively ovalbumin (OVA)-sensitized and -challenged model experiments, respectively. They were quarantined at the Animal Research Center of Kanazawa University. All animal procedures in this study conformed to the standards set in the Guidelines for the Care and Use of Laboratory Animals at the Takara-machi campus of Kanazawa University.

### Experimental protocol

#### Experiment 1: NO depletion by the NOS inhibitor L-NAME

**Figure 1:**
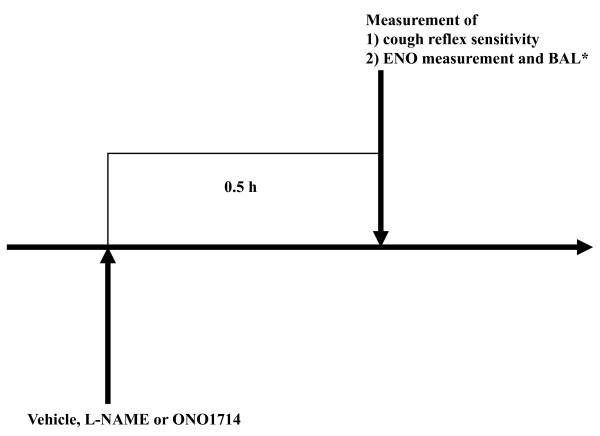
**Experimental design for the effect of NOS inhibitors in non-sensitized guinea pigs**. Vehicle (normal saline), the NOS inhibitor L-NAME solutions, or the iNOS inhibitor ONO1714 solutions were administered 0.5 h prior to cough reflex sensitivity to capsaicin and ENO measurement in non-sensitized guinea pigs weighing 500 to 550 g. *BAL was performed only in ONO1714 experiment.

**Figure 2:**
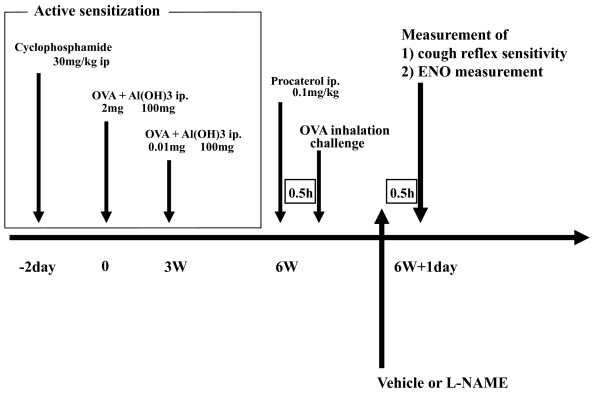
**Experimental design for the effect of L-NAME in OVA sensitized guinea pigs**. 24 h after OVA challenge, vehicle or L-NAME solutions were administered 0.5 h prior to cough reflex sensitivity to capsaicin and ENO measurement in OVA sensitized guinea pigs weighing 500 to 550 g.

The experimental protocol schemes are shown in Figure 1 and 2.

Non-sensitized and OVA-sensitized guinea pigs were divided into four groups: control (normal saline) group and groups receiving the non-selective NOS inhibitor L-NAME at doses of 3, 10, and 30 mg/kg (n = 6 for each group). L-NAME was dissolved in physiological saline. Vehicle (normal saline) and L-NAME solution were intraperitoneally administered 0.5 h prior to the measurement of cough reflex sensitivity to inhaled capsaicin.

#### Experiment 2: NO depletion by the selective iNOS inhibitor ONO1714

**Figure 3:**
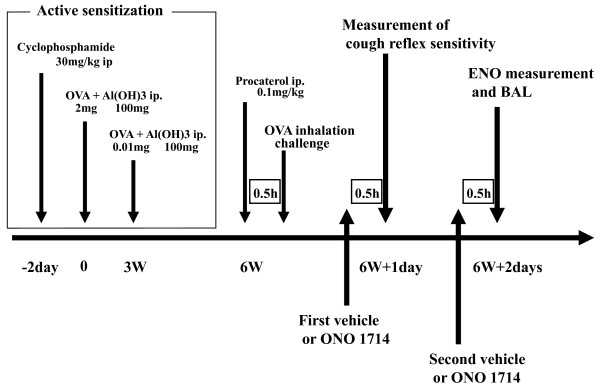
**Experimental design for the effect of ONO1714 in OVA sensitized guinea pigs**. 24 h after OVA challenge, vehicle or ONO1714 solutions were first administered 0.5 h prior to cough reflex sensitivity to capsaicin in OVA sensitized guinea pigs weighing 500 to 550 g, and again administered 0.5 h prior to ENO measurement and BAL, which was performed 24 h after the cough reflex sensitivity.

The experimental protocol schemes are shown in Figure 1 and 3.

Non-sensitized and OVA-sensitized guinea pigs were divided into three groups: control (normal saline) group and groups receiving the selective iNOS inhibitor ONO1714 at doses of 0.1 and 0.3 mg/kg (n = 6 for each group). ONO1714 (1S,5R,6R,7R)-7-chloro-3-imino-5-methyl-2-azabicyclo [4.1.0]heptane hydrochloride, a fused piperidine derivative, is a competitive iNOS inhibitor with a high safety margin (maximum tolerance dose (MTD) = 30 mg/kg, i.v., MTD/50% inhibitory dose for NOx accumulation = 3000 in mice) [21]; it was provided by Ono Pharmaceutical Co. Ltd. (Osaka, Japan). ONO1714 was dissolved in normal saline at doses of 0.1 and 0.3 mg/kg. Vehicle (normal saline) and ONO1714 solution were first intraperitoneally administered 0.5 h prior to the measurement of cough reflex sensitivity to inhaled capsaicin, and were again administered 0.5 h prior to ENO measurement followed by bronchoalveolar lavage (BAL), which was performed 24 h after the measurement of cough reflex sensitivity.

### Active sensitization and OVA challenge

Guinea pigs were OVA sensitized by the method reported by Muraki et al [22]. Guinea pigs weighing 150-200 g were intraperitoneally administered with 2.0 mg of OVA and 100 mg of aluminum hydroxide [Al(OH)_3_] two days after an intraperitoneal administration of 30 mg/kg cyclophosphamide. Three weeks later, boosting was performed by intraperitoneal administration of 0.01 mg of OVA and 100 mg of Al(OH)_3_. Three weeks after boosting, OVA sensitized guinea pigs were challenged with an aerosolized OVA solution under spontaneous breathing at 20 min after an intraperitoneal administration of diphenhydramine (20 mg/kg) to avoid acute anaphylactic respiratory distress. Conscious guinea pigs were placed in a dual chamber plethysmograph (head chamber volume, 1520 mL) (Model PMUA + SAR, Buxco Electronics, Sharon, CT, USA). Guinea pigs were challenged with 10 mg/mL OVA aerosol for 90 s (head chamber only, 0.08 mL/min output). The aerosol was generated using the DeVilbiss 646 nebulizer (DeVilbiss Co., Somerset, PA, USA) operated by compressed air at 7.57 L/min (Minipon 54B-588, Origin Medical Industry Co., Ltd., Tokyo, Japan). Cough reflex sensitivity to inhaled capsaicin was measured in the guinea pigs 24 h after OVA challenge.

### Cough reflex sensitivity to capsaicin

Cough reflex sensitivity was measured as described previously [23]. Capsaicin (30.5 mg) was dissolved in Tween 80 (1 mL) and ethanol (1 mL) and then dissolved in physiological saline (8 mL) to prepare a 10^-2 ^M stock solution, which was stored at -20°C. This solution was diluted with physiological saline to prepare 10^-6 ^and 10^-4 ^M solutions. Each conscious guinea pig was placed in an airtight, custom-built, transparent plastic box consisting of two isolated chambers: a head chamber (1600 mL volume) and a body chamber. Pressure in the body chamber was recorded. Cough was detected as a change in pressure (a rapid inspiration followed by rapid expiration). To disregard motion- and sneezing-related changes in pressure, movements of the guinea pigs were visually monitored. Cough reflex sensitivity of non-sensitized or OVA-sensitized guinea pigs was measured 0.5 h after treatment with vehicle, L-NAME, or ONO1714. Each guinea pig was initially exposed to the nebulized 10^-6 ^M capsaicin solution, followed by the 10^-4 ^M solution. Solutions were inhaled for 2 min with intervals of 8 min using the DeVilbiss 646 nebulizer operated by compressed air at 1.6 L/min (Iwaki Air Pump AP-115AN, Iwaki Co., Ltd., Tokyo, Japan). The nebulizer output was 0.037 mL/min. The number of coughs was counted during 2 min inhalation of each capsaicin solution and for additional 1 min. The total number of coughs during the 3-minute period was recorded for each concentration of capsaicin inhaled. These procedures were not performed in a blinded manner.

### Exhaled NO measurement

ENO was measured by the off-line method [24]. The expired air was sampled immediately after the measurement of cough reflex sensitivity in the L-NAME experiment and 24 h after the measurement (48 h after OVA challenge) in the ONO1714 experiment. Guinea pigs were intraperitoneally anesthetized with 75 mg/kg of sodium pentobarbital and were placed in a supine position. After tracheal cannulation with a polyethylene tube (outer diameter, 2.5 mm; inner diameter, 2.1 mm), the guinea pigs were artificially ventilated using a small animal respirator (Model 1680, Harvard Apparatus Co., Inc., South Natick, MA, USA) adjusted to a tidal volume of 10 mL/kg at a rate of 60 strokes/min. The expired air from the expiration valve of the ventilator was collected twice in the Mylar bag for 100 s. Within 2 h after sampling, the air was applied to the Sievers Model NOA280 chemiluminescence analyzer (Sievers Instruments, Boulder, CO, USA). The analyzer was calibrated with 650 ppb NO in N_2 _(Taiyo Nippon Sanso Corporation, Tokyo, Japan) according to the manufacturer's instructions. The sampling flow rate of the chemiluminescence analyzer was 200 mL/min.

### Bronchoalveolar lavage (BAL)

BAL was performed immediately after ENO sampling. The lungs were lavaged twice through the tracheal cannula with 10 mL of saline (total: 20 mL). The cells in BAL fluid (BALF) were stained with Turk's solution and counted in duplicate in a hemocytometer (in a Burker chamber). Differential cell counts were performed on a smear prepared using a cytocentrifuge and stained with Wright-Giemsa. In our previous study using the same experimental system, we have shown that augmentation of cough reflex sensitivity induced by single OVA inhalation is preserved during 48 h in OVA sensitized guinea pigs [23]. The procedures of anesthesia, tracheal cannulation, sampling of expired gas, and BAL require considerable time, which interferes with the measurement of cough reflex sensitivity that should be performed at scheduled time points. Therefore, we performed ENO and BAL cell component measurements at 24 h after the measurement of cough reflex sensitivity.

### Data analysis

Statistical differences were determined using the Student's *t*-test. A *P *value less than 0.05 was considered statistically significant.

## Results

**Figure 4:**
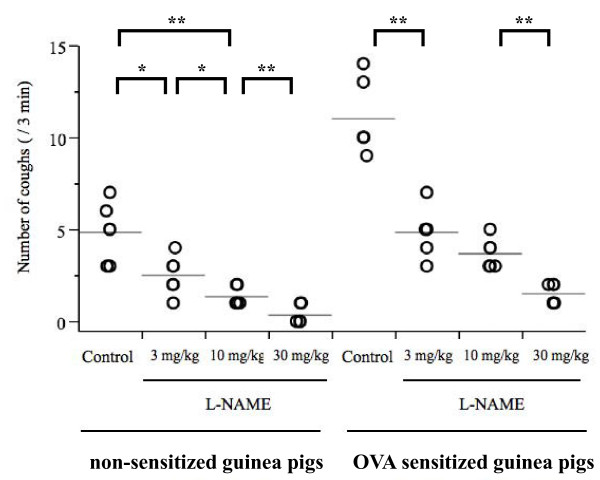
**Effect of L-NAME on cough reflex sensitivity, between non-sensitized and OVA-sensitized guinea pigs**. L-NAME suppressed cough reflex sensitivity to the 10^-4 ^M capsaicin solution in both groups dose-dependently. Each individual is presented in open circle, and the mean value is shown as a horizontal bar. n = 6 for each group, *p < 0.05 **p < 0.01

**Figure 5:**
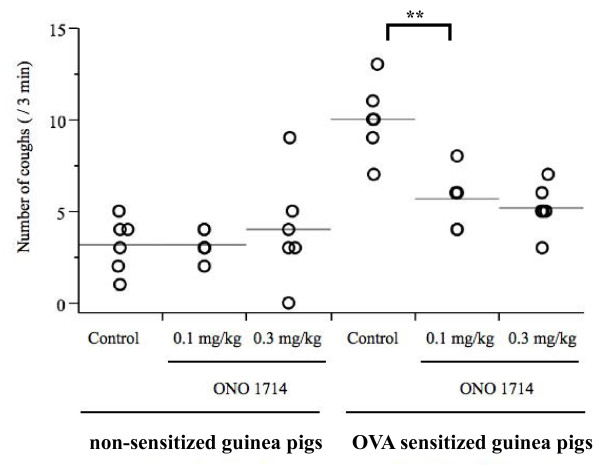
**Effect of ONO1714 on cough reflex sensitivity, between non-sensitized and OVA-sensitized guinea pigs**. In non-sensitized guinea pigs, there were no antitussive effects of ONO1714 observed, partial antitussive effect of ONO1714 on cough reflex sensitivity to the 10^-4 ^M capsaicin solution was observed in only OVA-sensitized guinea pigs. Each individual is presented in open circle, and the mean value is shown as a horizontal bar. n = 6 for each group, **p < 0.01

**Figure 6:**
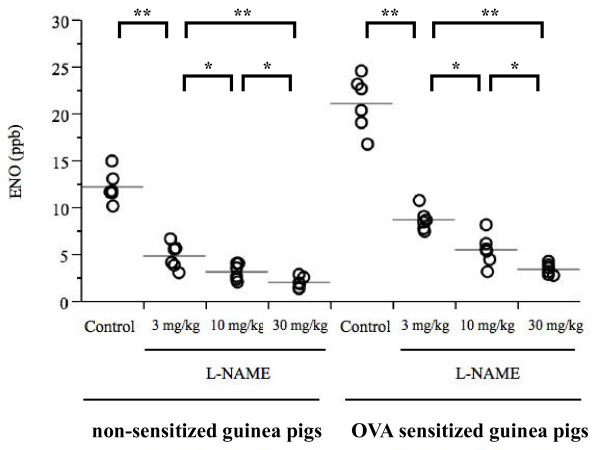
**Effect of L-NAME on ENO, between non-sensitized and OVA-sensitized guinea pigs**. L-NAME suppressed ENO in both groups dose-dependently. Each individual is presented in open circle, and the mean value is shown as a horizontal bar. n = 6 for each group, *p < 0.05 **p < 0.01

**Figure 7:**
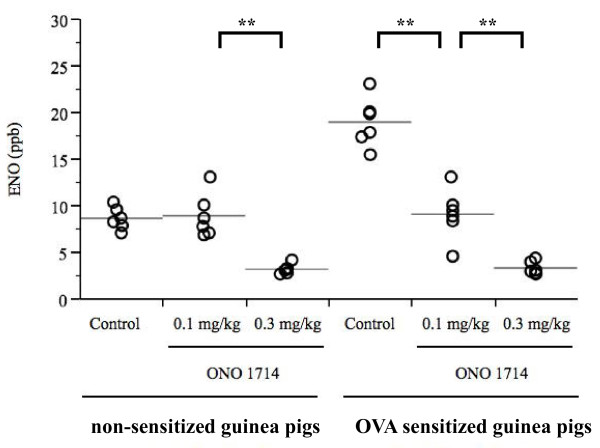
**Effect of ONO1714 on ENO, between non-sensitized and OVA-sensitized guinea pigs**. ONO1714 suppressed ENO in both groups. Its suppression was partial in non-sensitized guinea pigs and dose-dependently in OVA-sensitized guinea pigs. Each individual is presented in open circle, and the mean value is shown as a horizontal bar. n = 6 for each group **p < 0.01

Because the 10^-6 ^M capsaicin solution elicited few coughs in both non-sensitized and OVA-sensitized guinea pigs, cough reflex sensitivity was assessed by the number of coughs elicited by 10^-4 ^M capsaicin solution. Significantly (p < 0.0001) more frequent number of coughs was observed in OVA-sensitized guinea pigs compared to non-sensitized guinea pigs (Figure 4, Figure 5). ENO and eosinophil counts in BALF were significantly higher in OVA-sensitized guinea pigs compared to non-sensitized guinea pigs (Figure 6, Figure 7, Table 1), suggesting that the OVA-induced increase in cough reflex sensitivity may be associated with increased NO production and eosinophil accumulation.

**Table 1:** Bronchoalveolar lavage cell analysis

Total cells	(10^5 ^cells/mL)	Mac (%)	Neu (%)	Lym (%)	Eos (%)
non-sensitized guinea pigs					
Control	1.11 ± 0.11	71.8 ± 7.1	9.2 ± 5.6	1.8 ± 0.8	16.8 ± 5.6
ONO1714: 0.1 mg/kg	1.09 ± 0.12	74.5 ± 4.3	6.2 ± 1.5	1.4 ± 0.4	17.9 ± 4.7
ONO1714: 0.3 mg/kg	0.88 ± 0.09	76.0 ± 2.1	5.3 ± 3.3	1.5 ± 0.5	17.7 ± 1.6

OVA sensitized guinea pigs					
Control	2.63 ± 0.26	24.1 ± 3.1	2.5 ± 0.6	0.9 ± 0.3	72.4 ± 3.1
ONO1714: 0.1 mg/kg	2.68 ± 0.12	25.1 ± 1.0	2.8 ± 0.3	0.5 ± 0.2	71.6 ± 1.4
ONO1714: 0.3 mg/kg	2.64 ± 0.36	28.5 ± 3.6	0.8 ± 0.3	0.8 ± 0.3	69.9 ± 3.7

BAL was performed immediately after cough reflex sensitivity in non-sensitized guinea pigs. Whereas in OVA sensitized guinea pigs, BAL was performed 24 h after cough reflex sensitivity. MAC, macrophages; NEU, neutrophils; LYM, lymphocytes; EOS, eosinophils

### Experiment 1: NO depletion by the NOS inhibitor L-NAME

Figure 4 shows the effect of L-NAME on cough response to aerosolized capsaicin in non-sensitized and OVA-sensitized guinea pigs. The number of coughs induced by the 10^-4 ^M capsaicin solution was significantly suppressed by L-NAME in a dose-dependent manner in both non-sensitized and OVA-sensitized guinea pigs. ENO was also suppressed by L-NAME in both non-sensitized and OVA-sensitized guinea pigs (Figure 6).

### Experiment 2: NO depletion by the selective iNOS inhibitor ONO1714

Figure 5 shows the effect of ONO1714 on the coughs induced by the 10^-4 ^M aerosolized capsaicin solution in non-sensitized and OVA-sensitized guinea pigs. In non sensitized guinea pigs, the number of coughs was not influenced by ONO1714. On the other hand, in OVA-sensitized guinea pigs, the number of coughs was significantly suppressed, whereas inhibition by ONO1714 was not dose-dependent. Further, ENO was significantly suppressed by ONO1714 in both non-sensitized and OVA-sensitized guinea pigs (Figure 7), suggesting a discrepancy between inhibition of cough response to capsaicin and NO production in non-sensitized guinea pigs.

The effect of ONO1714 on BAL cell components s is shown in Table 1. Total cell and eosinophil counts were increased in OVA-sensitized guinea pigs compared to non-sensitized guinea pigs. ONO1714 had no influence on BAL cell components in any of the experimental guinea pigs.

## Discussion

The present study demonstrated that ENO from OVA-sensitized guinea pigs was significantly higher than that from non-sensitized guinea pigs; cough reflex sensitivity to inhaled capsaicin was significantly higher as well, as was shown in our previous study [23]. First, we performed NOS inhibition experiment utilizing the non-selective NOS inhibitor L-NAME, and clearly showed the antitussive effect caused by NO depletion with L-NAME, in both non-sensitized and OVA-sensitized guinea pigs. NO seemed to be a promoter in cough reflex sensitivity, and previous reports have pointed out the possibility of pro-inflammatory action of NO created by iNOS. Ohuchi et al [25] reported that iNOS induction by endotoxin inhalation enhanced substance P-induced microvascular leakage. Liu et al [26] examined the effects of OVA sensitization and challenge on iNOS gene expression in rats, and reported 3-fold increases of iNOS mRNA in their lungs sensitized to OVA alone. Therefore we considered that NO produced by iNOS might be a possible promoter in cough reflex sensitivity and performed the second experiment using the selective iNOS inhibitor ONO1714, which lowered ENO levels in both groups. ONO1714 reduced the OVA-induced increase in cough reflex sensitivity only in sensitized guinea pigs, but it did not affect the normal cough reflex sensitivity in non-sensitized guinea pigs. Our initial hypothesis emerged to be not supported by these findings,

Prado et al [27] showed that OVA sensitization enhanced the recruitment of iNOS-positive mononuclear cells and eosinophils in guinea pigs, and that L-NAME administration attenuated the number of mononuclear cells and eosinophils. Feder et al [28] reported that OVA-induced pulmonary eosinophilia in OVA-sensitized mice was significantly reduced by L-NAME, and they proposed an eosinophil chemotactic action of NO. Thus, we attempted to clarify the role of eosinophils, in addition to that of NO, as a promoter of cough reflex sensitivity. In this study, although BAL was not performed in the L-NAME experiment, ONO1714 despite administering twice was shown to have no inhibitory effect against BAL eosinophilia. We speculate that at least two pathways might be involved in cough reflex sensitivity; NO-dependent and eosinophil-dependent pathways.

Our results may be explained by an idea that L-NAME may work as a complete inhibitor of NO, while ONO1714 gives partial suppression of NO synthesis by exclusively inhibiting iNOS-dependent generation of NO. And we speculate the involvement of not only NO produced by iNOS, but also other crucial factors in cough reflex sensitivity. Several trials have explored whether or not NOS inhibition is effective in allergic bronchitis. In guinea pigs, which are antigen-induced chronic pulmonary inflammation models, cNOS inhibition causes bronchoconstriction and airway remodeling, and selective iNOS inhibition attenuates these effects [29]. These findings indicate that cNOS inhibition seems to be detrimental, and iNOS inhibition is a possible target of novel therapeutic agents in bronchial asthma. Disappointingly, Singh [30] reported that selective iNOS inhibition effectively reduced ENO, but did not alter airway hyperresponsiveness and inflammation. This is consistent with our present results that no anti-inflammatory effects were observed in selective iNOS inhibition with ONO1714. Corticosteroids, which are clinically the most effective medication for bronchial asthma, induce ENO depletion and suppression of airway eosinophilia, but have not been proved to diminish cough reflex sensitivity.

Pharmacological therapies for cough, such as codeine, have not made significant progress and the development of innovative antitussive agents is expected. Transient receptor potential cation channel, subfamily V, member 1(TRPV1) antagonists have been recognized as potential antitussives [31]. TRPV1 is a capsaicin receptor located in primary sensory neurons, where neuropeptides such as substance P are colocalized [32]. Moore et al [33] reported that an NO signaling cascade had unmasked functional neurokinin 2 receptors in acutely isolated nodose ganglion neurons of guinea pigs, and that NO seemed to act in afferent pathways of cough reflex. In this study, we could not explain the reason for partial inhibitory effect of iNOS inhibitor in cough reflex sensitivity. Therefore further studies involving other selective iNOS inhibitors or iNOS-knocked-out animals are needed.

## Conclusions

Our results showed that L-NAME totally suppressed cough reflex sensitivity to inhaled capsaicin and reduced ENO in both non-sensitized and OVA-sensitized guinea pigs. On the other hand, although ONO1714 showed a partial suppression of cough reflex sensitivity associated with further ENO suppression in OVA-sensitized guinea pigs, it had no antitussive effect despite ENO suppression in non-sensitized guinea pigs. ONO1714 did not influence BAL cell components 48 h after OVA challenge in sensitized animals. Our experiments suggest that NO is involved in both the normal cough reflex circuit and increased cough reflex sensitivity induced by allergic reaction. However, the pathway of involvement is too complex to be explained by the results of the present study. The precise mechanism of action need to be investigated in future studies.

## Competing interests

The authors declare that they have no competing interests.

## Authors' contributions

AH conceived the entire study, contributed to its design, helped in data acquisition and interpretation, and drafted the manuscript. MF conceived the study, contributed to its design, helped in data acquisition and interpretation. NO and AT participated in acquisition and interpretation of data. All authors read and approved the final manuscript.
